# Impact of Effort–Reward Imbalance and Burnout on the Compliance with Standard Precautions among Nurses and Midwives in Lebanese Hospitals

**DOI:** 10.3390/nursrep14020111

**Published:** 2024-06-11

**Authors:** Noha A. Sayrafi, Ali Salami, Ghassan Ghssein

**Affiliations:** 1 Faculty of Health Sciences, Beirut Arab University, Tareek Al Jadida, Afeef Al Tiba, Beirut 1105, Lebanon; nohasayrafi@hotmail.com; 2 Faculty of Sciences V, Lebanese University, Nabatieh 1700, Lebanon; a.salami@ul.edu.lb; 3 Faculty of Public Health, Islamic University of Lebanon (IUL), Khalde 30014, Lebanon

**Keywords:** effort–reward imbalance, burnout, standard precautions, healthcare-associated infections, healthcare workers, Lebanon

## Abstract

Background: Effort–reward imbalance (ERI) refers to the situation where there is a mismatch between the effort that healthcare workers (HCWs) put into their work and the rewards they receive in return. Burnout, on the other hand, is a psychological syndrome characterized by emotional exhaustion, depersonalization, and reduced personal accomplishment. This study aimed to assess the impact of ERI and burnout on the compliance with standard precautions (SPs) among nurses and midwives in Lebanese hospitals under the economic crisis and after the COVID-19 pandemic. Methods: Cross-sectional correlational study, based on self-administered questionnaire data, measuring the compliance with SPs, ERI, and burnout, in addition to the relationship between these factors, was performed among 409 nurses and midwives, working in Lebanese hospitals. Results: ERI was significantly associated with exposure to burnout among nurses and midwives, and burnout was found to be a significant predictor of nurses and midwives’ self-reported adherence with SPs. Conclusions: This study highlights the contribution of ERI and burnout to the chain of infection by decreased adherence to infection control SPs of nurses and midwives.

## 1. Introduction

Healthcare-associated infections (HAIs) are considered a major public health concern, affecting millions of patients worldwide each year. HAIs can result in prolonged hospital stays, increased antimicrobial resistance, financial burdens, and even excess deaths. Cross-contamination, often due to inadequate infection prevention measures, plays a significant role in HAI transmission [1,2,3,4].

To combat HAIs, standard precautions (SPs) are crucial. SPs involve a set of infection prevention practices aimed at protecting both healthcare workers (HCWs) and patients from the spread of infectious diseases. Key components of SPs include hand hygiene, the use of personal protective equipment (PPE), safe injection practices, transmission-based precautions, proper handling of contaminated equipment and surfaces, and respiratory hygiene [5,6,7].

By adhering to SPs, healthcare facilities can significantly reduce the risk of HAI transmission, thereby enhancing patient safety and improving overall healthcare outcomes [8,9].

HCWs should be familiar with and practice these precautions as part of their routine infection prevention and control measures, as they are often the first point of contact with patients [10,11], and their compliance with SPs is essential for preventing HAIs by reducing the risk of transmitting infectious agents to patients, as well as to other HCWs and visitors [12]. This not only protects patients from infections, but also helps to maintain a safe and healthy healthcare environment for everyone [13].

However, compliance with SPs is not always optimal, and there are several factors that can affect HCWs’ adherence to these practices [14]. One of the most important factors is knowledge and awareness because HCWs need to have a good understanding of the risks associated with the poor adherence to SPs. Lack of knowledge or misinformation can lead to confusion and noncompliance [15].

Another factor is workload and time constraints. HCWs are often under pressure to meet the demands of a heavy workload, which can make it challenging to follow all the necessary precautions. This can result in shortcuts being taken or steps being skipped, which can increase the risk of infection transmission. Adequate staffing levels and efficient work processes can help to alleviate some of this pressure and enable HCWs to prioritize infection prevention measures [15].

Environmental factors can also play a role in compliance. The physical layout of the healthcare facility, the availability of hand hygiene products, and the accessibility of PPE can all impact a HCW’s ability to follow SPs [16].

Individual factors such as attitudes, beliefs, and motivation can also influence compliance. HCWs who perceive infection control measures as burdensome or unnecessary may be less likely to follow them consistently. On the other hand, HCWs who understand the importance of these measures and feel a sense of personal responsibility for patient safety are more likely to comply [17].

Organizational culture and leadership can have a significant impact on compliance with SPs. A culture that prioritizes patient safety and infection control, and that provides clear guidance and support to HCWs, is more likely to foster a culture of compliance. Strong leadership that models and reinforces infection control practices can also help to promote adherence [9].

Effort–reward imbalance (ERI) and burnout are two important factors that can impact the compliance with SPs among HCWs in hospitals [18]. ERI refers to a mismatch between the effort that an individual puts into their work and the rewards they receive in return, which can lead to feelings of stress and dissatisfaction. Burnout, on the other hand, is a state of emotional, physical, and mental exhaustion caused by prolonged stress and overwork [19]. Both ERI and burnout have been found to have negative effects on the physical and mental health of HCWs, as well as on the quality of care they provide to their patients [20].

When it comes to compliance with SPs, both ERI and burnout can have significant implications. Research has shown that HCWs who experience high levels of ERI and burnout may be less likely to comply with SPs [20]. This may be due to a variety of factors, such as reduced motivation, decreased attention to detail, and increased risk-taking behavior. Additionally, HCWs who are experiencing ERI and burnout may be more likely to make errors or overlook important details, which can increase the risk of infection transmission [20].

Since 2019, Lebanon has been facing, in addition to the COVID-19 pandemic, a severe economic crisis, which has had a significant impact on all sectors of society, including healthcare. The country has been struggling with hyperinflation, a sharp devaluation of its currency, high unemployment rates, and a shortage of basic services [21,22].

The healthcare system in Lebanon has been severely affected by this economic crisis, in parallel with the COVID-19 pandemic, with many hospitals facing shortages of essential supplies, including medications, medical equipment, and fuel. This has resulted in an increased burden on HCWs, including nurses and midwives, who have been working long hours with limited resources to care for patients [23,24].

The economic crisis has also led to a significant decrease in the salaries of HCWs (including nurses and midwives). Many nurses have reported that their salaries have decreased by up to 80%, making it difficult for them to make ends meet [25]. This has resulted in an exodus of thousands of HCWs; for example, an estimated 15% of specialized nurses (neonatal intensive care unit) and 30% of midwives have left the country to seek better job opportunities abroad, further exacerbating the shortage of HCWs in Lebanon, which is seriously affecting the quality and accessibility of health [25].

The shortage of nurses and midwives has had a significant impact on the quality of care provided to patients in hospitals. They are often overworked and under-resourced, leading definitely to ERI and burnout among them, resulting to a decrease in compliance with SPs and a decrease in the quality of care they can provide [26,27].

This study aims to investigate the effect of ERI and burnout on the adherence to infection control SPs among nurses and midwives working in Lebanese hospitals. The impact of the current Lebanese economic crisis on the compliance of HCWs with SPs could be assessed too.

## 2. Materials and Methods

### 2.1. Study Design

Cross-sectional correlational study, based on self-administered questionnaire data, measuring the compliance with SPs, ERI, and burnout, in addition to the relationship between these factors.

### 2.2. Study Setting, Sampling, and Sample Size

The target population in this study are the nurses and midwives working in Lebanese hospitals. Stratified random sampling: Lebanon is divided into 8 governorates (Akkar, Baalback-Hermel, Beirut, Beqaa, Mount Lebanon, North Lebanon, Nabatieh, and South Lebanon). So, we divided the population into 8 strata (according to governorate). Then random selection was made for each stratum (governorate) to form the sample, and to make sure that all Lebanese governorates were represented in our study. 

As per last data of the World Bank (2018), Lebanon has 1.6 nurses and midwives per 1000 people (Lebanon—Nurses And Midwives—2023 Data 2024 Forecast 2001–2018 Historical, 2023), and as per last data of order of nurses (2021), 10,000 nurses registered in the order are working in hospitals, with the migration of almost 3500 nurses in the last years [26], so the estimated number of nurses and midwives working in hospitals will be around 9000. Using sample size calculator “Raosoft”, with a margin of error 5%, confidence level 95%, and response distribution 50%, the sample size must be 360 participants.

### 2.3. Power of the Study

As per our previous work [28], a prior statistical power analysis using GPower 3.1.9.2 software (Heinrich-Heine-Universität, Düsseldorf, Germany) revealed that the sample size n = 409 was enough to attain a statistical power of at least 80% with an alpha error of 5%, balanced on each side, and an effect size set to 0.2.

### 2.4. Inclusion and Exclusion Criteria

Nurses and midwives working in Lebanese hospitals (both men and women). Nurses and midwives working abroad retired or not working in hospitals and nurses working in administrative areas or in other nonclinical contexts were excluded from this study.

### 2.5. Instrument

A questionnaire developed and adopted from different studies to meet our study objectives was used, containing different parts:

Part A: Sociodemographic characteristics of participants, composed of 12 items, namely gender, age, marital status, educational status, profession, experience, working hours, unit of working, working in another job, type of hospital, distance between residence and working place per minutes, and type of transportation [29].

Part B: IPC knowledge and occupational exposure, composed of 6 items, namely frequency of IPC training received, experience of needle stick injury, experience of mucus membrane exposure to blood, hepatitis B immunization status, receiving IPC supportive supervisio,n and availability of IPC-related items [15].

Part C: Compliance with SPs [30,31,32], composed of 20-item scale that assesses the self-reported compliance with SPs, evaluating compliance with the use of PPE, disposal of sharps and wastes, decontamination of spills and used articles, and prevention of cross infection. Items will be measured using a 4-point Likert scale ranging “Never”, “Seldom”, “Sometimes”, and “Always”.

Part D: ERI [33,34], short version, composed of 16-item scale evaluating the 3 dimensions of the ERI model: extrinsic effort (measured by 3 items), extrinsic reward (measured by 7 items), and overcommitment (measured by 6 items). Items will be measured using a 4-point Likert scale ranging “Strongly Disagree”, “Disagree”, “Agree” and “Strongly Agree”.

Part E: Burnout [35,36], using Copenhagen Burnout Inventory, composed of 19-item scale evaluating 3 dimensions: Personal burnout (measured by 6 items), Work-related burnout (measured by 7 items) and Patient-related burnout (measured by 7 items). Items will be measured using a 5-point Likert scale ranging “Always”, “Often”, “Sometimes”, “Seldom”, and “Never”.

The questionnaire was generated in two versions: English and Arabic, independently translated by two bilingual Lebanese nationals. One was an official registered translator and the second was the main investigator, who is an expert in infection prevention and control; then, the main investigator synthesized the two Arabic versions to produce a single version, assuring the appropriate translation and cross-cultural adaptation.

Reliability analysis of the questionnaire items: The internal consistency of compliance with standard precautions, effort–reward imbalance, overcommitment, and burnout scales in this analysis as determined by Cronbach’s alpha were 0.893, 0.602, 0.610, and 0.929, respectively, indicating high reliability suitable for further analysis (Table 1).

### 2.6. Data Collection

The investigator gave out surveys (Kobo collect form), including an introduction explaining the study’s aims and privacy to the respondents. Participants who consented to take part answered independently the questions at a later date that was suitable for them so that the investigator could not affect the survey answer.

Data were collected in private and governmental Lebanese hospitals in the eight governorates in Lebanon between 1 April 2023 to mid-May 2023.

### 2.7. Data Analysis

Compliance with Standard Precautions (CSPS): For this information, Part C of the questionnaire was used [31,32,33]. The CSPS is a 20-item scale that assesses the self-reported compliance with SPs. The response set is a 4-point adjectival scale that consists of responses such as “never”, “seldom”, “sometimes”, and “always”. A score of 1 is interpreted as an “always” response, while 0 is applied for the other responses. A total range score of 0—20 is expected, with higher scores signifying better compliance with SPs. In addition, the compliance rate is also calculated (average compliance with the 20 items as a percentage). Questions 2, 4, 6 and 15 are negatively stated; thus, scores are reversed before computation [31].

Effort–Reward Imbalance (ERI): Short version was used in Part D of the questionnaire; see Table S1.

Effort scale: Effort is measured by three 4-point Likert-scaled items (items ERI1–ERI3) (Table S2) A total score based on the three items measuring effort varies between 3 and 12, as in (Table S3) [32].

Reward scale: Reward is measured by seven 4-point Likert-scaled items (items ERI4–ERI10), coded as in Table S2. A total score of these items varies between 7 and 28, as in Table S3. The lower the score, the fewer occupational rewards are supposed to be received by the person [33].

Overcommitment scale: Overcommitment is measured by six 4-point Likert-scaled items (items OC1–OC6) coded as in Table S2. A total score of these items varies between 6 and 24 as in Table S3 [33].

ER Ratio: To compute the ER-ratio, the effort score is put in the numerator and the reward score in the denominator: *ER* = *E*/R*c, where E is the effort score, R the reward score, and c a correction factor that adjusts for the unequal number of items of the effort and reward scores. For instance, if E contains 3 items and R contains 7 items, c, the correction factor is 3/7 = 0.428571. With this formulation, the interpretation of the ER-ratio is facilitated for descriptive purposes. For ER = 1, the person reports one effort for one reward, for ER < 1, there are fewer efforts for each reward, and for ER > 1, the person reports more efforts for each reward [33].

Burnout: Copenhagen Burnout Inventory (CBI) was used to measure the burnout among respondents [35]:

Personal burnout: From question 1 to question 6. Scoring: Always—100; Often—75; Sometimes—50; Seldom—25. Never/almost never—0. Total score on the scale is the average of the scores on the items. If fewer than three questions have been answered, the respondent is classified as nonresponder [35].

Work-related burnout: Question 7 to question 13. Reversed score for last question (question 13). Scoring: Always—100; Often—75; Sometimes—50; Seldom—25. Never/almost never—0. Total score on the scale is the average of the scores on the items. If less than four questions have been answered, the respondent is classified as nonresponder [35].

Patient-related burnout: Question 14 to question 19. Scoring: Always—100; Often—75; Sometimes—50. Seldom—25. Never/almost never—0. Total score on the scale is the average of the scores on the items. If less than three questions have been answered, the respondent is classified as nonresponder [35].

Once respondents have completed all of the items, their scores are calculated by averaging the scores for each item within each domain. The scores for each domain can range from 0 to 100, with higher scores indicating higher levels of burnout [35]. A summary score over the midpoint (‘50’) classified the individual as exposed to burnout [20].

### 2.8. Statistical Analysis

The SPSS software (IBM Corp., Released 2013, SPSS Statistics for Windows Version 22.0, Armonk, NY, USA) was utilized for both statistical analyses and data management/cleaning purposes. The Kolmogorov–Smirnov test was employed to assess the normality distribution of quantitative variables.

Descriptive statistics were presented in the form of frequencies and percentages for categorical variables, while means (±) standard deviation (SD) were used to report normally distributed continuous variables. When the variable deviated considerably from normality, it was presented as the median and interquartile range (IQR). To examine the differences in compliance with standard precautions based on sociodemographic and work-related factors, the Mann–Whitney U and Kruskal–Wallis tests were employed. Multiple comparisons on continuous variables were adjusted using the Bonferroni method.

To assess the internal consistency of the measurement tools, Cronbach’s α was utilized. Spearman’s rho correlation was implemented to explore the correlation between the main variables. Furthermore, a multiple linear regression analysis was performed to identify any correlations between compliance with standard precautions, which was the dependent variable, and various factors such as ER ratio, overcommitment scale (OC), and CBI burnout score (including personal-related, work-related, and patient-related burnouts). All statistical analyses were conducted with a significance level of *p* < 0.05.

## 3. Results

### 3.1. I. Healthcare Workers Sociodemographic and Work-Related Characteristics

Four hundred and nine respondents returned filled questionnaires, out of which 66% were women with a mean age of 34.03 ± 7.29 years, and 239 (58.4%) of them being married. A bachelor’s degree was common 207 (50.6%), while 230 (56.2%) were working as registered nurses. More than 227 (55%) respondents had less than 10 years of healthcare experience, the average years of healthcare experience among registered nurses was 11.39 ± 6.70 years. The majority were full-time and overtime workers: 309 (75.7%), with more than 40 working hours per week (average working hours per week was 47.12 ± 15.27), while just 70 (17.2%) reported that they had another job. A significant proportion of the respondents worked in medical/surgical wards: 170 (41.6%), while 78 (19.1%) worked in intensive care units, and 67 (16.4%) in obstetrics and gynecology wards. The respondents who reported that they had previously attended infection control training were 157 (38.4%). A total of 290 (70.9%) respondents had been vaccinated for Hepatitis B. Respondents who reported that they had been experienced needle stick injury and mucus membrane exposure were 185 (45.2%) and 138 (33.7%), respectively, while 181 (44.3%) respondents reported that they had been received IPC supportive supervision during the work (Table 2).

### 3.2. ERI, Burnout, and Compliance with SP Scores

The mean ERI and burnout scores for the study population were (1.05) and (37.31), respectively. Despite the mean score falling below the thresholds indicative of exposure to burnout, (37.31%) of HCWs were above the burnout exposure limit (score > 50/100), and (54.03%) experienced a negative ERI (ER Ratio > 1). With a maximum possible score of 20, the mean SP compliance score was 12.00 ± 5.31, which was occupied by (67.2%) of HCWs. The lowest percentage of compliance was obtained for the reuse of masks and other disposable PPEs (18.2%). The percentage of compliance was higher for performing hand washing between patients’ contact (75.7%). Concerning the recapping of needles after use, 241 (59.4%) nurses and midwives responded “never” for this practice, but a considerable number performed this activity “always” or “sometimes”: 141 (34.7%). Regarding item 5, almost half of them (221, 54.2%) disposed of sharps in appropriate containers. As for the item that corresponds to the act of showering in cases of extensive splashes, even when the professional has used PPE, 256 (63.5%) responded “always”. Regarding the use of a surgical mask or a combination with goggles and apron whenever there is a possibility of splashes or spills, 295 (72.3%) indicated the alternative “always”, followed by 88 (21.6%) for “sometimes”. In the item for the use of an apron/coat when they are exposed to blood, body fluids, or any excretion from patients, 258 (63.7%) always performed this practice. A total of 301 (74.1%) changed gloves between contacts with patients, revealing a weak compliance regarding the use of gloves, and 292 (72.1%) nurses and midwives reported that they always sanitize their hands immediately after removing the gloves. With respect to the item about the reuse of surgical mask or disposable PPE, 224 (55%) of nurses and midwives said they “never” perform this activity and 74 (18.2%) do perform it. Concerning item 2, using only water for hand washing, only 60 (15%) responded “never”; however, 232 (57.9%) said “always” for this practice, reflecting a very weak awareness about the appropriate method of hand hygiene. The use of alcohol-based products to sanitize the hands, as an alternative in case they are not visibly dirty, resulted in 175 (42.8%) responses for the option “always”. In case of decontamination of surfaces and equipment after use, 291 (71.7%) nurses and midwives “always” performed this activity, followed by 85 (20.9%) for “sometimes”. With regard to item 17, 27 (67.3%) responded “always” in waste segregation, and 288 (71.7%) said they “always” cleaned surfaces immediately after spilling blood or other body fluids. As for the disposal of the sharps bin, 195 (47.8%) responded that they “always” empty it only when it is full, 134 (32.8%) responded with “sometimes”, and just 34 (8.3%) responded “never”. The distribution of adherence, burnout, and ERI scores by demographic factors are shown in Table 3.

### 3.3. Differences in Compliance to Standard Precautions by Sociodemographic and Work-Related Characteristics among Nurses and Midwives

The post hoc Bonferroni analysis revealed notable variations in compliance with SPs among nurses and midwives of different age groups. Specifically, the compliance rate was higher among nurses and midwives under 22 years old than those aged between 22–25 and 26–30 years old (*p* = 0.001 and *p* = 0.002, respectively). Similarly, nurses and midwives over 31 years old showed higher compliance with standard precautions than those aged between 26 and 30 years old (*p* = 0.008). The Mann–Whitney U test indicated that nurses and midwives who had previously received IPC training exhibited lower compliance with standard precautions than those without prior knowledge (*p* = 0.025). Conversely, nurses and midwives who had been vaccinated against Hepatitis B, experienced occupational exposure, and received IPC supportive supervision at work demonstrated higher compliance with standard precautions (*p* < 0.001, *p* < 0.001, *p* < 0.001, respectively) (Table 4).

### 3.4. Correlation between Measured Variables

Spearman’s rho correlation was implemented to explore the correlation between the main variables (Table 5). Table 5 shows the values of Spearman’s rho correlation analysis.

### 3.5. Multiple Linear Regression Analysis Predicting Compliance to Standard Precautions

A multiple regression model was run to predict compliance to standard precautions from ER ratio, overcommitment Scale (OC), and burnout (personal-related, work-related, and patient-related) scores. These variables statistically significantly predicted compliance with standard precautions, F (5, 403) = 10.235, *p* < 0.001.

CBI personal burnout positively impacts compliance with standard precautions (0.032 [*p* = 0.030]). Moreover, CBI patient-related burnout has a negative effect on compliance with standard precautions (−0.092 [*p* < 0.001]).

## 4. Discussion

According to this study, over half (54.03%) of the nurses and midwives studied had experienced ERI, while (37.31%) had been exposed to burnout. The mean burnout score for Lebanese nurses and midwives in this study (M = 37.31405) fall within the range of previous studies conducted in European countries (M = 17.5–46.5). The reported ERI score of (1.056461) exceeds the range found in those same studies (M = 0.57–0.82) [20]. When compared to other studies among nurses—Polish (ERI = 0.78, burnout = 39.7), Slovakian (ERI = 0.70, burnout = 45.4), and Ecuadorian (ERI = 0.71, burnout = 41.5)—the Lebanese burnout scores were found to be closest to those obtained by the Polish study, while the ERI score remained the highest among all mentioned studies [19]. The closest score to the Lebanese ERI score was found in a German study among physicians and nurses (ERI = 1.33) [37].

Regarding these findings, the prevalence of burnout among Lebanese nurses and midwives was comparable to that reported in the Ecuadorian study at (35.8%). However, the percentage of Lebanese nurses and midwives who experienced ERI was significantly higher at (21%) [20].

The high prevalence of ERI among Lebanese nurses and midwives could be related to the Lebanese economic crisis, which has led to a devaluation of the currency; as a result, many Lebanese HCWs had experienced significant financial hardships [28]. This high prevalence of ERI among Lebanese nurses and midwives can be attributed to several factors, including low salaries, inadequate working conditions, and an overwhelming workload. Nurses and midwives in our study had reported working long hours with little rest and inadequate compensation (75.7% of them reported working more than 40 working hours per week with an average of 47.12 SD ± 15.27 h per week), leading to a sense of unfairness and frustration.

In this study, results show that (32.8%) of nurses and midwives were not complying with SPs, while the overall compliance with SPs was found to be (67.2%), compared to (44.2%) of Ecuadorian nurses and (71.0%) of Canadian nurses reporting adherence to SPs [19]. This finding is in line with one study performed in Ethiopia, where (65%) of HCWs complied with SPs [16], while another Ethiopian study showed that the compliance with SPs was found to be very low (12%) [38]. This difference between studies might be due to the differences in sociodemographic factors, cultural factors, and accessibility to adequate supply of equipment related to infection prevention and control, and since Lebanon has been grappling with a severe economic crisis, leading to a shortage of basic supplies and services including medical and paramedical supplies, this could be an attributable factor.

In this study, when each of the specific components of SPs was analyzed, the better results have been observed in some of the items. For example, HCWs were found to be always compliant with washing hands between patients (75.7%), washing hands immediately after removal of gloves (72.1%), wearing clean gloves whenever there is a possibility of exposure to body fluids (72.3%), changing gloves between contacts with different patients (74.1%), and appropriate use of face masks (76.5%). Better findings had been found in one Ethiopian study in which 92.2% of HCWs were found to be always compliant with washing hands after body fluid exposure, 80.6% washed hands immediately after removal of gloves, 88.7% wore clean gloves whenever there was a possibility of exposure to body fluids, 88.9% changed gloves between contacts with different patients, and 87.2% placed used sharps in puncture-resistant containers at point of use [38].

When looking at the overall adherence to SPs, it was discovered that nurses and midwives are not fully implementing these measures in their practice, which puts them at higher risk. The results of percentage of compliance per item showed that there were failures in compliance in several areas such as hand hygiene, inappropriate use of PPE and improper handling of sharp materials. These findings are consistent with a study conducted in Brazil [39].

According to our study, nurses and midwives who are younger than 22 years old (who have recently graduated) tend to have a higher compliance rate with SPs than other age groups (*p* = 0.001). This finding is consistent with an earlier study conducted in Ethiopia, which showed that fresh graduates were twice as likely to comply with SPs practices [16]. This could be attributed to their recent learning, strong dedication, and fear of communicable diseases and HAIs. On the other hand, nurses and midwives over 31 years of age in our study exhibited a higher compliance rate with SPs than those within the age groups of 22 to 30 (*p* = 0.008). This finding contradicts the Ethiopian study and could be due to the fact that HCWs had attended more workshops, seminars, and training sessions that cover infection prevention and control topics, encouraging compliance with SPs and better work practices for safer work environment [16].

In this study, it had been shown that there is no statistically significant correlation between burnout scores and sociodemographic characteristics. This finding is consistent with previous studies published on the topic. Past research conducted on various nursing populations around the world had yielded inconsistent results regarding the relationship between sociodemographic factors and burnout [20,40].

In addition, this study had shown that there is a statistically significant correlation between ERI score and personal, work-related, and patient-related burnout scores among nurses and midwives (*p* < 0.001, *p* < 0.001, and *p* < 0.001, respectively). This correlation suggests that when HCWs have an ERI score that is high, they are more likely to experience burnout in personal, work-related, and patient-related areas. This finding is in line with many studies conducted in Ecuador, China, and Ethiopia [19,20,26]. One possible cause of this correlation is stress. HCWs who experience high levels of stress due to an imbalance between their effort and the rewards they receive are more likely to struggle with burnout. Additionally, HCWs who feel that they are not being valued or rewarded appropriately for their hard work are also more likely to experience burnout. Another possible cause of this correlation is a lack of support. HCWs who feel unsupported in their work, whether it is from colleagues or management, may experience a greater sense of burnout [19,41,42].

On the other hand, the study had shown that there is a statistically significant correlation between CSPS and ERI, work-related, and patient-related burnout scores (*p* = 0.028, *p* = 0.004 and *p* < 0.001, respectively). Higher levels of burnout (including emotional exhaustion, depersonalization, and reduced personal accomplishment) have been associated with decreased adherence to recommended infection prevention and control practices. One possible explanation for this correlation is that work-related burnout can lead to a sense of disengagement from one’s work, which may result in decreased attention to important details such as hand hygiene, wearing PPEs, and following cleaning and disinfecting protocols for example [8,17,43]. In addition, burnout can result in reduced empathy and emotional exhaustion, which may make it more challenging for HCWs to maintain motivation and energy needed to adhere to SPs consistently. Patient-related burnout, which refers to the emotional exhaustion and depersonalization that result from working with challenging patients or experiencing high levels of stress, may also contribute to decreased compliance with SPs [44,45].

No statistically significant correlation was found in this study between over-commitment score and CSPS. There are multiple possible causes of this absence of correlation. First, it is possible that the measures of overcommitment and compliance were not sensitive enough to detect differences between individuals. Secondly, it could be that other factors, such as job satisfaction, overall work environment or even ERI and burnout exposure assessed in our study, are more influential in determining an individual’s likelihood to comply with SPs. Lastly, there may be an interaction between overcommitment and situational factors, such as stress or workload, that also play a role in determining compliance with SPs [12,45].

The multiple linear regression assessing the ability of ERI, personal, work-related, and patient-related burnouts to predict the compliance with SPs. The results had been shown that these variables are statistically significantly predicted compliance with SPs, F (5, 403) = 10.235, *p* < 0.001. Patient-related burnout was found to be a unique incremental predictor of adherence to SPs (β = −0.092, *t* = −5.823, *p* < 0.01), negatively affected the compliance with SPs. This finding is in line with many studies conducted; for example, an Ecuadorian study reported that burnout was found to be a unique incremental predictor of adherence to infection control precautions [20]. This might be related to daily dealing with difficult patients, because the latter can be a major source of stress for HCWs, which can contribute to burnout and a reduced ability to comply with SPs. First, the challenging behaviors such as aggression, noncompliance, and verbal abuse that can be particularly stressful for HCWs, such behaviors can make it difficult for HCWs to provide care and follow SPs. Secondly, the high workload, because patient who require a high level of care, such as those with complex medical needs or those who are critically ill, can place a heavy workload on HCWs. This can lead to fatigue and stress, which can increase the risk of burnout and reduce compliance with SPs [12,46].

The evidence suggests that burnout is a significant and independent predictor of nurses and midwives’ self-reported compliance with SPs. This indicates that burnout may increase the likelihood of noncompliance, which in turn could lead to an increase in HAIs, transmissible occupational infections, and higher rates of absenteeism [19]. Ultimately, this could have a negative impact on the provision of safe and accessible healthcare for all.

## 5. Limitations

The limitations of this study include the fact that cross-sectional data design makes it impossible to draw conclusions about causality. Then, both dependent (compliance with standard precautions) and independent variables (ERI and burnout) were assessed using self-reported surveys, increasing the study’s susceptibility to subject reporting bias.

## 6. Conclusions and Recommendations

ERI and burnout are important factors that can negatively impact the compliance with standard precautions among HCWs. When the latter experience high levels of work demand without adequate rewards and recognition, they may become emotionally exhausted and depersonalized, and experience reduced personal accomplishment. This can lead to burnout, which has been found to be associated with a decreased adherence to SPs.

This is a serious concern, as failure to comply with SPs can lead to HAIs, which can have serious consequences for both patients and HCWs. Therefore, it is crucial for healthcare organizations to address the issue of ERI and burnout among HCWs, and to implement strategies that support and promote their wellbeing, so that they can provide safe and effective care of their patients while protecting themselves from the risk of infection.

Based on the available evidence, there are several recommendations that healthcare organizations can consider to address this issue:

1. Addressing the root causes of ERI by providing HCWs with adequate resources, support, and recognition to help them cope with work demands.
2. Urging the healthcare organizations and both syndicates of nurses and midwives to take action to adjust the salaries of nurses and midwives in order to support their crucial work and ensure their financial stability, because in Lebanon, they are not being adequately compensated for their efforts. Low salaries are not only a financial burden for them, but also hinder the quality of care that can be provided to patients. So, we need to ensure that they are being paid fairly and equitably for the important work they do.
3. Developing and implementing effective interventions to reduce burnout among HCWs. This can include measures such as providing opportunities for rest and recovery, promoting work–life balance, and offering mental health support.
4. Providing regular training and education on the importance of adhering to standard precautions and ensuring that HCWs have access to the appropriate equipment and supplies needed to comply.
5. Fostering a culture of safety and accountability within healthcare organizations, where adherence to standard precautions is seen as a core responsibility of all HCWs.
6. Monitoring compliance with SPs and providing feedback to HCWs on their performance, with a focus on recognizing and reinforcing positive behaviours.
7. Investing in research to better understand the relationship between effort–reward imbalance, burnout, and compliance with standard precautions, and to identify interventions that are effective in promoting adherence to these precautions among HCWs.

By implementing these recommendations, healthcare organizations can help to reduce the risk of HAIs and promote the wellbeing of HCWs, while ensuring that patients receive safe and effective care.

## Figures and Tables

**Table 1:** Reliability analysis of the questionnaire items.

Variables	Cronbach α
Compliance with standard precautions	0.893
Effort–reward imbalance (ERI)	0.602
Overcommitment (OC)	0.610
Copenhagen Burnout Inventory (CBI)	0.929

**Table 2:** Sociodemographic and work-related characteristics of participants (n = 409).

Variable	Category	n (%)	Mean ± SD
Gender	Women	270 (66.0)	
	Men	139 (34.0)	
Age	<22	5 (1.2)	34.032 ± 7.290
	22–25	40 (9.8)	
	26–30	113 (27.6)	
	≥31	251 (61.4)	
Marital status	Divorced	9 (2.2)	
	Married	239 (58.4)	
	Single	159 (38.9)	
	Widowed	2 (0.5)	
Educational level	BP	4 (1.0)	
	BT	31 (7.6)	
	TS	26 (6.4)	
	LT	58 (14.2)	
	BS	207 (50.6)	
	Master’s degree	83 (20.3)	
Total years of experience	<5	52 (12.7)	11.391 ± 6.704
	5–9	175 (42.8)	
	10–14	117 (28.6)	
	≥15	65 (15.9)	
Current position	Midwife	77 (18.8)	
	Nurse aid	60 (14.7)	
	Nursing supervisor	42 (10.3)	
	Registered nurse	230 (56.2)	
Areas of practice	Emergency department	51 (12.5)	
	Intensive care unit	78 (19.1)	
	Medical ward	125 (30.6)	
	ObGyn	67 (16.4)	
	Surgical ward	45 (11.0)	
	Others	43 (10.5)	
Working hours per week *	≤40	99 (24.3)	47.117 ± 15.266
	>40	309 (75.7)	
Having another job *	No	337 (82.8)	
	Yes	70 (17.2)	
Prior experience in infection and prevention control (IPC) education	No	252 (61.6)	
	Yes	157 (38.4)	
Prior occupational exposure (needle stick)	No	224 (54.8)	
	Yes	185 (45.2)	
Prior occupational exposure (mucus membrane exposure)	No	271 (66.3)	
	Yes	138 (33.7)	
Hepatitis B immunization status	No	119 (29.1)	
	Yes	290 (70.9)	
Receiving IPC supportive supervision during the work	No	228 (55.7)	
	Yes	181 (44.3)	

* Missing value. n: sample size. SD: standard deviation.

**Table 3:** Distribution of adherence, burnout, ERI scores by demographic factors.

Factors	n (%)	ERI Ratio Mean (SD)	Burnout Mean (SD)	Adherence Mean (SD)
Gender				
Women	270 (66.0)	1.048 (0.380)	37.405 (18.377)	12.096 (5.244)
Men	139 (34.0)	1.073 (0.455)	37.137 (17.084)	11.813 (5.445)
Age				
<22	5 (1.2)	1.294 (0.612)	41.627 (25.176)	17.4 (1.673)
22–25	40 (9.8)	1.166 (0.480)	45.203 (16.366)	10.575 (5.358)
26–30	113 (27.6)	1.066 (0.408)	38.043 (21.122)	10.965 (5.405)
≥31	251 (61.4)	1.030 (0.387)	35.643 (16.104)	12.586 (5.161)
Civil status				
Divorced	9 (2.2)	1.054 (0.462)	42.614 (17.404)	10.667 (5.315)
Married	239 (58.4)	1.044 (0.371)	36.405 (17.681)	12.059 (5.171)
Single	159 (38.9)	1.079 (0.454)	38.206 (18.358)	11.949 (5.552)
Widowed	2 (0.5)	0.765 (0.202)	51.191 (9.821)	-
Education				
BP	4 (1.0)	1.293 (0.633)	59.177 (13.767)	15.5 (0.577)
BT	31 (7.6)	1.058 (0.409)	36.124 (17.111)	10.548 (6.577)
TS	26 (6.4)	1.01 (0.371)	36.699 (13.296)	12.231 (5.279)
LT	58 (14.2)	1.086 (0.401)	38.802 (18.308)	13.5 (4.139)
BS	207 (50.6)	1.065 (0.409)	37.097 (18.587)	11.536 (5.674)
Master’s degree	83 (20.3)	1.019 (0.408)	36.400 (17.430)	12.410 (4.401)
Profession				
Midwife	77 (18.8)	1.054 (0.311)	31.316 (15.556)	11.636 (5.579)
Nurse aid	60 (14.7)	1.047 (0.409)	37.698 (16.259)	11.533 (5.9502)
Nursing supervisor	42 (10.3)	1.167 (0.509)	40.131 (18.910)	13.286 (4.697)
Registered nurse	230 (56.2)	1.039 (0.413)	38.707 (18.565)	12.009 (5.137)
Experience years				
<5	52 (12.7)	1.172 (0.501)	45.295 (19.029)	11.673 (5.324)
5–9	175 (42.8)	1.000 (0.393)	34.394 (17.348)	12.154 (5.443)
10–14	117 (28.6)	1.041 (0.369)	35.494 (16.335)	12.179 (5.328)
≥15	65 (15.9)	1.143 (0.400)	42.066 (18.826)	11.523 (4.959)
Having another job				
No	337 (82.8)	1.102 (0.396)	36.077 (17.982)	12.080 (5.229)
Yes	70 (17.2)	1.238 (0.400)	43.049 (16.533)	11.529 (5.727)
Unit of working				
Emergency department	51 (12.5)	1.109 (0.459)	35.578 (15.059)	12.588 (4.867)
Intensive care unit	78 (19.1)	1.079 (0.421)	36.284 (18.869)	12.474 (5.386)
Medical ward	125 (30.6)	1.028 (0.428)	38.645 (17.722)	11.752 (5.510)
ObGyn	67 (16.4)	1.039 (0.303)	32.184 (14.999)	11.388 (5.918)
Surgical ward	45 (11.0)	1.066 (0.407)	42.584 (19.393)	12.489 (4.985)
Others	43 (10.5)	1.051 (0.406)	39.849 (20.867)	11.605 (4.425)

**Table 4:** Differences in compliance to standard precautions by sociodemographic and work-related characteristics of participants (n = 409).

Variable	Category	Mean ± SD MD (Q_1_–Q_3_)	Multiple Comparisons (Bonferroni Correction)	*p*
Gender	Women	12.096 ± 5.244 13.00 (8.00–16.00)	-	0.814 ^
	Men	11.813 ± 5.444 14.00 (7.00–16.00)	-	
Age	<22 ^a^	17.400 ± 1.673 17.00 (16.00–18.00)	a > b (*p* = 0.001)	<0.001 ^^
	22–25 ^b^	10.575 ± 5.358 11.50 (5.00–14.00)	a > c (*p* = 0.002)	
	26–30 ^c^	10.965 ± 5.405 13.00 (7.00–15.00)	d > c (*p* = 0.008)	
	≥31 ^d^	12.586 ± 5.161 14.00 (9.00–16.00)	-	
Marital status	Divorced	10.667 ± 5.315 12.00 (7.00–15.00)	-	0.748 ^^
	Married	12.059 ± 5.171 13.00 (8.00–16.00)	-	
	Single	11.950 ± 5.552 13.00 (7.00–16.00)	-	
	Widowed	-	-	
Educational level	BP	15.500 ± 0.577 15.50 (15.00–16.00)	-	0.179 ^^
	BT	10.548 ± 6.577 13.00 (4.50–16.50)	-	
	TS	11.896 ± 5.516 14.00 (7.00–16.00)	-	
	LT	13.500 ± 4.139 14.50 (12.00–16.00)	-	
	BS	11.536 ± 5.674 13.00 (7.00–16.00)	-	
	Master’s degree	12.231 ± 5.279 13.00 (10.00–15.00)	-	
Total years of experience	<5	11.673 ± 5.324 13.00 (7.00–16.00)	-	0.605 ^^
	5–9	12.154 ± 5.443 14.00 (8.00–16.00)	-	
	10–14	12.180 ± 5.328 14.00 (8.00–16.00)	-	
	≥15	11.523 ± 4.960 13.00 (8.00–15.00)	-	
Current position	Midwife	11.636 ± 5.580 12.00 (7.00–17.00)	-	0.642 ^^
	Nurse aid	11.533 ± 5.950 14.00 (6.00–16.00)	-	
	Nursing supervisor	13.286 ± 4.697 14.00 (11.00–16.00)	-	
	Registered nurse	12.009 ± 5.137 13.00 (8.00–16.00)	-	
Areas of practice	Emergency department	12.588 ± 4.867 14.00 (10.50–16.00)	-	0.733 ^
	Intensive care unit	12.474 ± 5.386 14.00 (9.00–16.00)	-	
	Medical ward	11.752 ± 5.510 14.00 (7.00–16.00)	-	
	ObGyn	11.388 ± 5.918 12.00 (6.50–17.00)	-	
	Surgical ward	12.489 ± 4.985 14.00 (11.00–16.00)	-	
	Others	11.605 ± 4.425 12.00 (8.50–15.50)	-	
Working hours per week	≤40	11.535 ± 4.676 13.00 (8.00–15.00)	-	0.109 ^
	>40	12.142 ± 5.501 14.00 (8.00–16.00)	-	
Having another job	No	12.080 ± 5.229 14.00 (8.00–16.00)	-	0.380 ^
	Yes	11.529 ± 5.727 13.00 (8.00–16.00)	-	
Prior experience in IPC education	No	12.464 ± 5.289 14.00 (9.00–16.50)	-	0.025 ^
	Yes	11.255 ± 5.271 13.00 (7.00–15.00)	-	
Prior occupational exposure (needle stick)	No	10.924 ± 5.226 12.00 (7.00–15.50)	-	<0.001 ^
	Yes	13.303 ± 5.124 14.00 (12.00–17.00)	-	
Prior occupational exposure (mucus membrane exposure)	No	11.679 ± 5.116 13.00 (8.00–16.00)	-	0.064 ^
	Yes	12.630 ± 5.632 14.00 (8.00–17.00)	-	
Hepatitis B immunization status	No	9.740 ± 5.720 9.00 (5.00–15.50)	-	<0.001 ^
	Yes	12.928 ± 4.843 14.00 (10.00–16.00)	-	
Receiving IPC supportive supervision during the work	No	10.289 ± 5.436 11.00 (6.00–15.00)	-	<0.001 ^
	Yes	14.155 ± 4.270 15.00 (13.00–17.00)	-	

^ Mann–Whitney U test. ^^ Kruskal–Wallis test. n: sample size. SD: standard deviation. *p*: *p*-value.

**Table 5:** Correlation between measured variables.

Variables	ER Ratio	OC Scale	CBI Personal Burnout	CBI Work-Related Burnout	CBI Patient-Related Burnout	CSPS
ER ratio	1					
OC scale	0.358 (*p* < 0.001)	1				
CBI personal burnout	0.438 (*p* < 0.001)	0.232 (*p* < 0.001)	1			
CBI work-related burnout	0.465 (*p* < 0.001)	0.271 (*p* < 0.001)	0.588 (*p* < 0.001)	1		
CBI patient-related burnout	0.492 (*p* < 0.001)	0.262 (*p* < 0.001)	0.449 (*p* < 0.001)	0.608 (*p* < 0.001)	1	
CSPS	−0.109 (*p* = 0.028)	Ns	Ns	−0.144 (*p* = 0.004)	−0.307 (*p* < 0.001)	1

ER: effort–reward. CBI: Copenhagen Burnout Inventory. CSPS: compliance with standard precautions scale. *p*: *p*-value. Ns: not significant.

## Data Availability

The data presented in this study are available on request from the corresponding author.

## Supplementary Materials

The following supporting information can be downloaded at: https://www.mdpi.com/article/10.3390/nursrep14020111/s1, Table S1. ERI-Questionnaire. Short version. Item coding; Table S2. 4-point Likert scale answer format in the ERI-Questionnaires; Table S3. ERI-Questionnaire. Short version. Construction of scores; Full Version of the Questionnaire

## Public Involvement Statement

Nurses and midwives currently working at Lebanese hospitals participated in this study.

## Guidelines and Standards Statement

This manuscript was drafted against the STROBE criteria for reporting cross-sectional studies.
