# Supplementary material for: Impact of Effort–Reward Imbalance and Burnout on the Compliance with Standard Precautions among Nurses and Midwives in Lebanese Hospitals
Source: Nurs Rep. 2024 Jun 11;14(2):1477–93. doi: 10.3390/nursrep14020111 (PMC11206807; doi:10.3390/nursrep14020111)
Supplement: Supplementary file 1 [file nursrep-14-00111-s001.zip › nursrep-2989752-supplementary.pdf]

Supplementary Materials

Table S1. ERI-Questionnaire. Short version. Item coding

		Strongly disagree	Disagree	Agree	Strongly agree
ERI1	I have constant time pressure due to a heavy workload.				
ERI2	I have many interruptions and disturbances while performing my job.				
ERI3	Over the past few years, my job has become more and more demanding.				
ERI4	I receive the respect I deserve from my superior or a respective relevant person.				
ERI5	My job promotion prospects are poor. **(Reverse coding)**				
ERI6	I have experienced or I expect to experience an undesirable change in my work situation. **(Reverse coding)**				
ERI7	My job security is poor. **(Reverse coding)**				
ERI8	Considering all my efforts and achievements, I receive the respect and prestige I deserve at work.				
ERI9	Considering all my efforts and achievements, my job promotion prospects are adequate.				
ERI10	Considering all my efforts and achievements, my salary / income is adequate.				
OC1	I get easily overwhelmed by time pressures at work				
OC2	As soon as I get up in the morning, I start thinking about work problems.				
OC3	When I get home, I can easily relax and 'switch off' work. **(Reverse coding)**				
OC4	People close to me say I sacrifice too much for my job				
OC5	Work rarely lets me go, it is still on my mind when I go to bed.				
OC6	If I postpone something that I was supposed to do today I'll have trouble sleeping at night				

Table S2. 4-point Likert scale answer format in the ERI-Questionnaires.

Strongly disagree	□ **(1)**
Disagree	□ **(2)**
Agree	□ **(3)**
Strongly agree	□ **(4)**

Table S3. ERI-Questionnaire. Short version. Construction of scores

Scales	Items	Range
Effort scale	ERI 1 to ERI 3	3 to 12
Reward scale	ERI 4 to ERI 10	7 to 28
Overcommitment scale	OC 1 to OC 6	6 to 24

Full Version of the Questionnaire

A. Socio-demographic Characteristics:

1. Gender:
   □ Female □Male
2. Age:
   □<20 years □20-30 years □30-40 years □>40 y
3. Marital status:
   □Single □Married □Divorced □Widowed
4. Educational status:
   □BP □BT □TS □LT □BS □Master's degree
5. Profession:
   □Nurse aid □Registered nurse □Midwife □Nurse supervisor
6. Years of experience:
   □<5 years □5-10 years □10-15 years □15-20 years □>20 years
7. Working hours per week:
   □<40 hours □40-46 hours □>46 hours
8. Unit of working: ___________
9. Having another job: □Yes □No
10. Did you receive any training on Infection Control Standard Precautions?
    □Yes □No

B. Compliance with Standard Precautions:

1. I wash my hands between patient contacts:
   □Never □Seldom □Sometimes □Always
2. I only use water for hand washing:
   □Never □Seldom □Sometimes □Always
3. I use alcohol hand rubs as an alternative if my hands are not visibly soiled:
   ☐Never ☐Seldom ☐Sometimes ☐Always
4. I recap used needles after giving an injection:
   ☐Never ☐Seldom ☐Sometimes ☐Always
5. I put used sharp articles into sharps boxes:
   ☐Never ☐Seldom ☐Sometimes ☐Always
6. The sharps box is only disposed when it is full:
   ☐Never ☐Seldom ☐Sometimes ☐Always
7. I remove PPE in a designated area:
   ☐Never ☐Seldom ☐Sometimes ☐Always
8. I take a shower in case of extensive splashing even after I have put on PPE:
   ☐Never ☐Seldom ☐Sometimes ☐Always
9. I cover my wound(s) or lesion(s) with waterproof dressing before patient contacts:
   ☐Never ☐Seldom ☐Sometimes ☐Always
10. I wear gloves when I am exposed to body fluids, blood products, and any excretion of patients:
    ☐Never ☐Seldom ☐Sometimes ☐Always
11. I change gloves between each patient contact:
    ☐Never ☐Seldom ☐Sometimes ☐Always
12. I decontaminate my hands immediately after removal of gloves:

☐Never ☐Seldom ☐Sometimes ☐Always

13. I wear a surgical mask alone or in combination with goggles, face shield, and apron whenever there is a possibility of a splash or splatter:
    ☐Never ☐Seldom ☐Sometimes ☐Always
14. My mouth and nose are covered when I wear a mask:
    ☐Never ☐Seldom ☐Sometimes ☐Always
15. I reuse mask or disposable PPE:
    ☐Never ☐Seldom ☐Sometimes ☐Always
16. I wear a gown or apron when exposed to blood, body fluids, or any patient excretions:
    ☐Never ☐Seldom ☐Sometimes ☐Always
17. Waste contaminated with blood, body fluids, secretion, and excretion are placed in red plastic bags irrespective of patient's infective status:
    ☐Never ☐Seldom ☐Sometimes ☐Always
18. I decontaminate surfaces and equipment after use:
    ☐Never ☐Seldom ☐Sometimes ☐Always
19. I wear gloves to decontaminate used equipment with visible soils:
    ☐Never ☐Seldom ☐Sometimes ☐Always
20. I clean up spillage of blood or other body fluid immediately with disinfectants:
    ☐Never ☐Seldom ☐Sometimes ☐Always

C. ERI:

1. I have constant time pressure due to a heavy workload:
   ☐Strongly disagree ☐Disagree ☐Agree ☐Strongly agree
2. I have many interruptions and disturbances while performing my job:
   ☐Strongly disagree ☐Disagree ☐Agree ☐Strongly agree
3. Over the past few years, my job has become more and more demanding:
   ☐Strongly disagree ☐Disagree ☐Agree ☐Strongly agree
4. I receive the respect I deserve from my superior or a respective relevant person:
   ☐Strongly disagree ☐Disagree ☐Agree ☐Strongly agree
5. My job promotion prospects are poor:
   ☐Strongly disagree ☐Disagree ☐Agree ☐Strongly agree
6. I have experienced or I expect to experience an undesirable change in my work situation:
   ☐Strongly disagree ☐Disagree ☐Agree ☐Strongly agree
7. My job security is poor:
   ☐Strongly disagree ☐Disagree ☐Agree ☐Strongly agree
8. Considering all my efforts and achievements, I receive the respect and prestige I deserve at work:
   ☐Strongly disagree ☐Disagree ☐Agree ☐Strongly agree
9. Considering all my efforts and achievements, my job promotion prospects are adequate:
   ☐Strongly disagree ☐Disagree ☐Agree ☐Strongly agree
10. Considering all my efforts and achievements, my salary / income is adequate:
    ☐Strongly disagree ☐Disagree ☐Agree ☐Strongly agree
11. I get easily overwhelmed by time pressures at work:
    ☐Strongly disagree ☐Disagree ☐Agree ☐Strongly agree
12. As soon as I get up in the morning, I start thinking about work problems:
    ☐Strongly disagree ☐Disagree ☐Agree ☐Strongly agree

13. When I get home, I can easily relax and ‘switch off’ work:
☐Strongly disagree ☐Disagree ☐Agree ☐Strongly agree
14. People close to me say I sacrifice too much for my job:
☐Strongly disagree ☐Disagree ☐Agree ☐Strongly agree
15. Work rarely lets me go, it is still on my mind when I go to bed:
☐Strongly disagree ☐Disagree ☐Agree ☐Strongly agree
16. If I postpone something that I was supposed to do today I’ll have trouble sleeping at night:
☐Strongly disagree ☐Disagree ☐Agree ☐Strongly agree

D. Burnout:

1. How often do you feel tired?
□Always □Often □Sometimes □Seldom □Never
2. How often are you physically exhausted?
☐Always ☐Often ☐Sometimes ☐Seldom ☐Never
3. How often are you emotionally exhausted?
☐Always ☐Often ☐Sometimes ☐Seldom ☐Never
4. How often do you think: “I can’t take it anymore”?
☐Always ☐Often ☐Sometimes ☐Seldom ☐Never
5. How often do you feel worn out?
☐Always ☐Often ☐Sometimes ☐Seldom ☐Never
6. How often do you feel weak and susceptible to illness?
☐Always ☐Often ☐Sometimes ☐Seldom ☐Never
7. Is your work emotionally exhausting?
☐Always ☐Often ☐Sometimes ☐Seldom ☐Never
8. Do you feel burnt out because of your work?
☐Always ☐Often ☐Sometimes ☐Seldom ☐Never
9. Does your work frustrate you?
☐Always ☐Often ☐Sometimes ☐Seldom ☐Never
10. Do you feel worn out at the end of the working day?
☐Always ☐Often ☐Sometimes ☐Seldom ☐Never
11. Are you exhausted in the morning at the thought of another day at work?
☐Always ☐Often ☐Sometimes ☐Seldom ☐Never
12. Do you feel that every working hour is tiring for you?
☐Always ☐Often ☐Sometimes ☐Seldom ☐Never
13. Do you have enough energy for family and friends during leisure time?
☐Always ☐Often ☐Sometimes ☐Seldom ☐Never
14. Do you find it hard to work with clients?
☐Always ☐Often ☐Sometimes ☐Seldom ☐Never
15. Do you find it frustrating to work with clients?
☐Always ☐Often ☐Sometimes ☐Seldom ☐Never
16. Does it drain your energy to work with clients?
☐Always ☐Often ☐Sometimes ☐Seldom ☐Never
17. Do you feel that you give more than you get back when you work with clients?
☐Always ☐Often ☐Sometimes ☐Seldom ☐Never
18. Are you tired of working with clients?
☐Always ☐Often ☐Sometimes ☐Seldom ☐Never

19. Do you sometimes wonder how long you will be able to continue working with clients?
☐Always ☐Often ☐Sometimes ☐Seldom ☐Never
